# Spatial Variation of Key Bioclimatic Factors in the Natural Range of *Picea koraiensis* and Implications for Its Conservation

**DOI:** 10.3390/biology15171465

**Published:** 2026-08-28

**Authors:** David Kombi Kaviriri, Tianyi Liu, Ling Yang

**Affiliations:** 1 College of Forestry, Beijing Forestry University, Beijing 100083, China; davidkaviriri@yahoo.fr; 2 Department of Natural Resources Management, Faculty of Agricultural Sciences, University of Kinshasa, Kinshasa XI 023, Democratic Republic of the Congo; 3 College of Forestry, Northeast Forestry University, Harbin 150040, China

**Keywords:** *Picea koraiensis*, natural range, conservation, bioclimatic factors

## Abstract

*Picea koraiensis* (Korean spruce) is a boreal tree species of considerable ecological and economic importance in Northeast Asia, particularly within its natural range in China, Russia and the Korean Peninsula. This study investigated spatial variation in key bioclimatic factors and their relationships with terrain gradients to clarify how these variables influence the species’ distribution. Using high-resolution climate data and species-occurrence records, we examined the effects of elevation, slope and aspect on temperature and precipitation patterns across the species’ range. Precipitation-related variables, including bio13, bio14 and bio15, were closely associated with habitat suitability, whereas elevation and slope affected local temperature stability and moisture availability. Higher elevations and steeper slopes generally provided cooler, wetter conditions favorable for *P. koraiensis*. Climate modelling predicts that suitable habitats will shift northwards and to higher elevations in response to climate change and are likely to contract under high-emission scenarios. Identifying climate refugia and areas with stable bioclimatic conditions provides important insights for conservation prioritization. This study highlights the need for targeted conservation strategies, including protecting identified climate refugia, maintaining or restoring connectivity corridors between suitable habitats, and carefully assessing assisted migration to climatically favorable northern and higher-elevation areas, to support the long-term viability of the species under rapid climate change.

## Simple Summary

*Picea koraiensis* (Korean spruce) is an important tree species in forests across Northeast Asia, including China, Russia and the Korean Peninsula. In this study, we sought to understand how local climatic conditions—especially temperature and rainfall—vary across the landscape and how this variation affects the Korean spruce growing space. We found that rainfall is the most important factor for the spruce’s survival conditions. However, topography also matters: higher elevations and steeper slopes create cooler, wetter conditions that benefit *P. koraiensis*. Using future climate models, we predict that suitable growing areas will shift northwards and to higher altitudes as the planet warms. If greenhouse gas emissions remain high, these habitats may shrink substantially. By identifying areas where the climate will remain stable, we help conservation efforts. Our results will guide managers in designing targeted actions, such as protecting key habitats and assisting the Korean spruce to establish in new areas, to ensure that the species survives in a rapidly changing climate.

## 1. Introduction

The boreal tree species *Picea koraiensis* (Korean spruce) is a key conifer in the temperate forests of Northeast Asia, occurring in China, Russia, and the Korean Peninsula. As a long-lived canopy tree, it contributes to forest carbon storage through the accumulation of woody biomass and its role in maintaining forest structure. It also contributes to local microclimatic and hydrological regulation and provides habitat within coniferous forest ecosystems. In addition to these ecological functions, *P. koraiensis* has economic value for timber production, silviculture, and ecological restoration in Northeast Asia. Forests in Northeast China provide important ecosystem services, have shown measurable responses to climatic variation, and have experienced substantial anthropogenic land-cover disturbance [1,2,3]. These regional pressures may increasingly affect suitable habitats and populations of *P. koraiensis* across parts of its natural range.

Within the framework of niche theory, the geographical distribution of a species reflects the portion of environmental space that permits its persistence, although the realized distribution may also be constrained by dispersal, biotic interactions, and historical factors. Climatic envelope theory provides a practical basis for linking species-occurrence records to the climatic conditions associated with suitable habitats. Under climate change, range-shift ecology predicts that species may track suitable climatic conditions through poleward and elevational movements, unless such movements are constrained by habitat fragmentation or limited dispersal capacity [4,5]. At the local scale in northeastern China, climatic variability has been shown to influence the biological diversity and functioning of mixed forests [6]. For *P. koraiensis*, this indicates that temperature, precipitation, and seasonality are relevant environmental dimensions to consider when evaluating its distribution. Understanding the spatial variation in these factors across the natural range of the species is essential for developing conservation strategies that are responsive to current and future climatic conditions. Previous research has emphasized the importance of bioclimatic stability for the persistence of boreal tree species, particularly in regions undergoing rapid climate change [7]. Such changes are expected to alter habitat suitability, potentially causing distributional shifts, local extinctions, or the emergence of newly suitable areas [4].

*P. koraiensis* has a broad geographical distribution that encompasses diverse environmental conditions. Climate-based distribution models, including CLIMEX and MaxEnt, have been widely reviewed in South Korea as tools for evaluating species distributions under environmental change [8]. Examining the spatial variation in bioclimatic factors, including mean annual temperature, seasonal precipitation, and temperature extremes, is therefore important for understanding the ecological responses of the species along environmental gradients [9].

A comprehensive understanding of bioclimatic variation across the natural range of *P. koraiensis* is essential for its effective conservation. By examining spatial variation in temperature, precipitation, and seasonal climatic patterns, researchers can identify current and potential future habitats conducive to the persistence of the species under changing climatic conditions. Such analyses can also identify areas that may function as climate refugia, which could be important for the long-term survival of populations during periods of environmental change.

Species distribution models (SDMs) and bioclimatic analyses can help identify areas at risk of habitat loss, as well as potential future suitable habitats and dispersal routes. For *P. koraiensis*, mapping current and future distributions is necessary to develop conservation strategies that protect not only the species itself but also the ecological functions it supports. Such strategies may include identifying potential migration routes, maintaining connectivity between suitable habitats, and prioritizing regions for reforestation or active management. Accurate bioclimatic information is therefore essential for developing effective conservation measures, including habitat protection, assisted migration where appropriate, and the regulation of human activities in climatically sensitive areas.

A review of the literature on spatial bioclimatic variation indicates that populations located at the margins of a species’ range are generally more vulnerable to climatic change than those in central areas because they often occur under less favorable environmental conditions [10]. Regional assessments of conifer dieback in Siberia and the Far East indicate that climatic anomalies, including changes in temperature and moisture, can interact with biotic factors to affect forest health [11]. More broadly, translating species-distribution information into practical climate-change adaptation remains challenging because projected changes must be interpreted alongside dispersal, management, and ecological uncertainty [12]. These considerations highlight the need to assess bioclimatic variation at appropriate spatial scales in order to identify vulnerable and resilient populations [6,13].

The aim of this study is to identify areas within the natural range of *P. koraiensis* that could serve as future climate refugia, where relatively stable conditions may support the continued persistence of the species. To achieve this aim, the study maps and analyzes the spatial distribution of key bioclimatic variables across the species’ natural range and examines their relationships with terrain gradients. We tested three hypotheses: (H1) habitat suitability of *P. koraiensis* is more closely associated with precipitation-related bioclimatic variation than with other climatic factors; (H2) elevation and slope modify local temperature and moisture conditions, thereby contributing to spatial heterogeneity in habitat suitability; and (H3) future climate change will shift suitable habitats northwards and towards higher elevations, while areas with relatively stable bioclimatic conditions will serve as potential climate refugia. This spatial analysis provides a basis for developing management strategies to mitigate climate-change impacts, guide reforestation efforts, and prioritize conservation measures in areas where the species is most likely to persist in the long term. The present study therefore bridges ecological understanding and conservation practice by clarifying how spatial bioclimatic variation influences the distribution dynamics and resilience of *P. koraiensis*.

## 2. Materials and Methods

### 2.1. Study Area and Recording the Occurrence Data of Picea koraiensis

**Figure 1:**
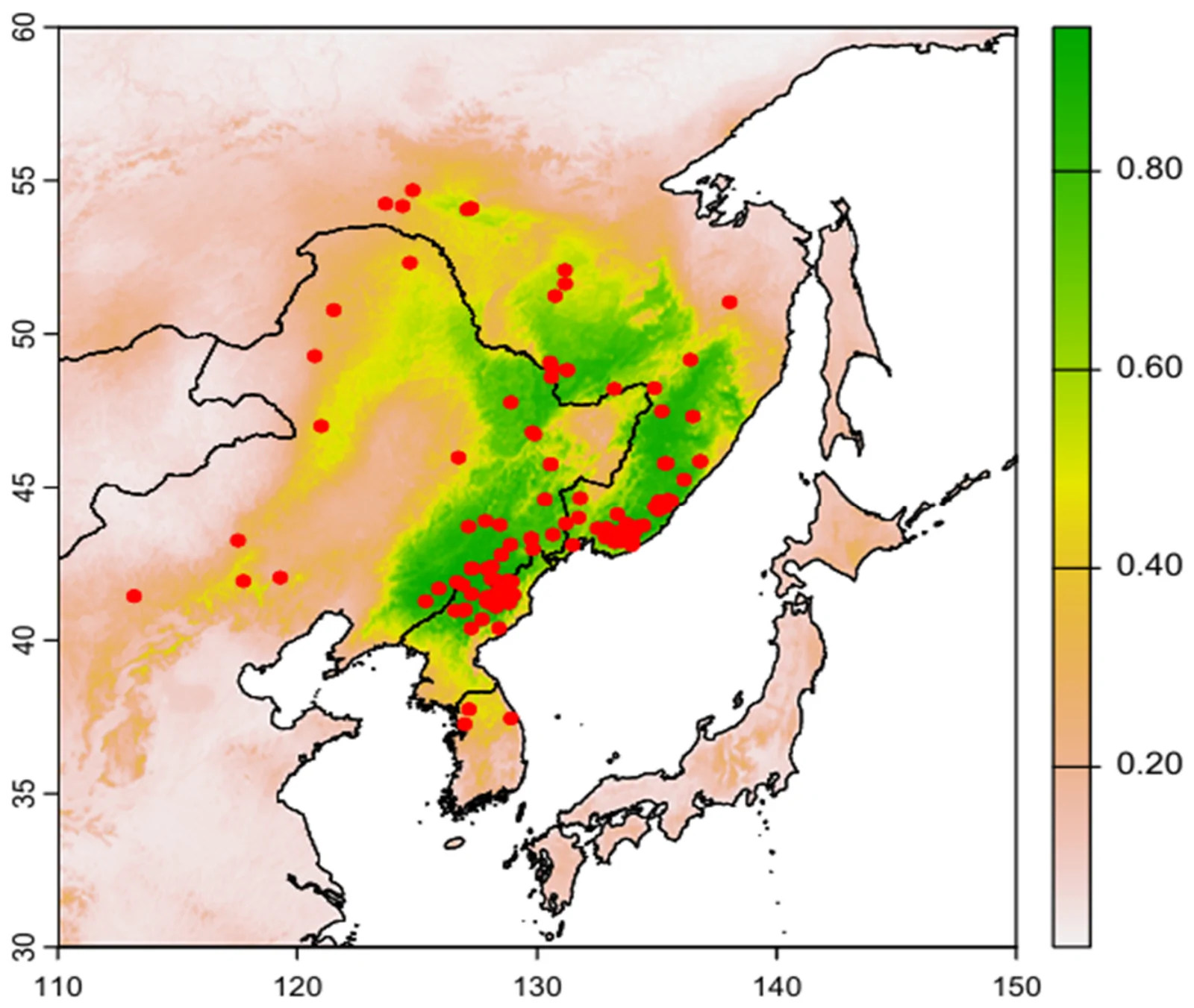
Distribution of *Picea koraiensis* according to the probability of current occurrence in its natural range. Red dots indicate the geographic coordinates of occurrence records analyzed in this study.

The natural range of *P. koraiensis* extends across the temperate regions of north-eastern China (Heilongjiang, Jilin and Liaoning provinces), the Korean peninsula and the Russian Far East, especially the southern parts of the Primorsky region. It typically grows in mountainous areas and valleys with cool, moist conditions and prefers well-drained soils at altitudes of 400 to 2200 m above sea level. The geographical limit of the species’ range extends to around 52° N in the Russian Far East and reaches as far as 38° N in north-east China and North Korea. Its north-eastern limit is about 134° E in the Russian Far East, while its north-western extent reaches 124° E in Heilongjiang Province, China. The study area covers the entire distribution range of *P. koraiensis* from 110° E to 150° E and from 30° N to 60° N (Figure 1). In this geographic range, *P. koraiensis* coexists with a variety of tree species that predominate in temperate mixed forests, including *Pinus koraiensis*, *Abies holophylla* (Manchurian fir), *Quercus mongolica* (Mongolian oak), *Tilia amurensis* (Amur lime), *Ulmus japonica* (Japanese elm), and *Betula* spp. More broadly, forest types and their associated soils form mosaics of coniferous and deciduous vegetation adapted to contrasting environmental conditions [14]. Different climatic conditions prevail in the forests of this region, with temperature differences ranging from −6.57 °C per 100 m in the coldest areas to 13.21 °C in the warmer regions in southwest and central China. Annual rainfall ranges from 315 mm in the driest areas to 1371 mm in the wettest regions. The altitude of *P. koraiensis* occurrences varies between 24 m and 735 m above sea level.

The geographical coordinates for the occurrence of *P. koraiensis* were obtained from the Global Biodiversity Information Facility (GBIF) (available online at https://doi.org/10.15468/dl.7552pf, accessed on 14 October 2024) and the Chinese Virtual Herbarium (CVH, https://www.cvh.ac.cn, accessed on 26 August 2024) [15,16]. These databases provided locality data, which were verified and corrected by taxonomic processing and literature searches. GBIF contains 581 records of occurrences, while CVH provides 216 images of specimens for verification of species, including varieties. All georeferenced points were used to extract environmental, climatic and terrain data. To improve the accuracy of the data, Google Earth 7.0 was used to visualize patterns and exclude points in non-forest areas. Duplicate records were removed using the R package “dplyr”version 1.2.1. Occurrence data were managed using the version 1.3-16 of dismo package [17]. The bioclimatic data were standardized to a spatial resolution of 5 km^2^ (2.5′) for consistency. After filtering, the final dataset comprised 122 unique occurrence points for analysis.

### 2.2. Environmental Variables Selection

In order to analyze the distribution evolution of *P. koraiensis*, historical, current and future climate data were obtained from the WorldClim database (https://www.worldclim.org/, accessed on 21 October 2024). The historical climate data included the last glacial maximum (LGM, ~22,000 years ago) and the middle Holocene (MH, ~6000 years ago). These data came from the CCSM4 and MIROC-ESM models [18,19]. The current climate data covered the period from 1970 to 2000 and came from WorldClim version 2.1 (accessed on 21 October 2024). For the future projections, the SSP2-4.5 and SSP5-8.5 scenarios were used for the periods from 2041 to 2060 and from 2081 to 2100, respectively [20]. The datasets, comprising 19 bioclimatic variables at a resolution of 2.5 arcminutes, also included elevation data from WorldClim, with slope and aspect derived using ArcGIS 10.5. In the absence of historical and future terrain data, it was assumed that elevation, slope and aspect would remain constant. Due to the large number of bioclimatic variables and possible multicollinearity, we performed a correlation analysis using the “corrr” package to identify a reduced set of relatively uncorrelated environmental variables. To further ensure robustness, we performed principal component analysis (PCA) to assess the contribution of these variables and assessed multicollinearity using the “vifstep” function. The final nine variables (bio2, bio3, bio5, bio13, bio14, bio15, aspect, slope, and elevation) were then used in the version 1.2-67 of sdm modelling framework [21] (Figure S1, Table S1).

### 2.3. Modelling Spatial Future Changes

To project past and predict future spatial distributions of *P. koraiensis*, an ensemble model was developed using three powerful algorithms (‘rf’, ‘svm’, and ‘maxent’) from the 22 methods available in the sdm package (Table S2) [21]. The package also generated 124 pseudo-occurrence points within the study area to complement known occurrences. Model parameters were optimized using the standard method in sdm. Occurrence data were split 70:30 for training and validation, with equal weighting of presence and pseudo-occurrence data to ensure balanced results.

Each algorithm was run for 10 repetitions, resulting in a total of 440 simulations across all data partitions. Model performance was evaluated using area under the curve (AUC) and true skill statistic (TSS); TSS was included because it provides a prevalence-independent measure of model discrimination [22]. Only simulations meeting the study-defined score threshold of 0.7 were used in the final ensemble model. The ensemble model was then applied to environmental data from different time periods to predict the distribution of *P. koraiensis*. This included assessing climatic stability from the Last Glacial Maximum (LGM) to future scenarios (2081–2100), identifying stable habitats, and assessing potential colonization or extinction risks.

### 2.4. Statistical Analysis and Impact Assessment

Model performance was evaluated using area under the curve (AUC) and true skill statistic (TSS). AUC values range from 0.5 (random prediction) to 1 (perfect prediction), and TSS was interpreted following Allouche et al. [22]. A minimum TSS value of 0.41 was adopted in this study to ensure model robustness. The “getEvaluation” function of the sdm package was used to determine the relative importance of the environmental variables and generate response curves explaining the distribution of *P. koraiensis* [21]. The “gui” function automated parameterization and visualization of the species’ responses to environmental factors. To account for uncertainties, different modelling techniques and climate scenarios were compared to obtain a comprehensive understanding of the current and future distribution of *P. koraiensis*. Potential distribution maps were created, and suitable areas were quantified using the study-defined threshold of 0.41, with the results converted to square kilometers. Predicted distributions across different time periods were overlaid to identify permanently suitable habitats that are likely to persist under all scenarios and to highlight areas at risk of colonization or extinction.

The selected key environmental variables were subjected to descriptive statistical analysis and mapped with color gradients to illustrate climatic variability. To gain insight into the influence of topography on local climate within the distribution area of *P. koraiensis*, relationships between climatic variables and topographic factors were determined. General relationships among the variables were assessed using Pearson correlation. Multiple linear regression analyses and generalized additive models (GAMs) were used to quantify these relationships and account for possible non-linear effects. An altitude-band analysis was performed to elucidate scaling relationships between climate and altitude. In addition, spatial autocorrelation analysis was performed to analyze climatic patterns within the distribution area. Global Moran’s I was used to assess whether patterns between occurrence points and selected bioclimatic variables showed clustering, dispersion, or randomness. Local Indicators of Spatial Association (LISA) were used to identify regions where data values were strongly positively or negatively associated with one another within the distribution area. Three latitudinal and longitudinal gradients were identified to determine whether geographical gradients influenced variation in bioclimatic factors. Bioclimatic variables within the distribution range of *P. koraiensis* were compared. Together, these analyses characterized spatial and temporal variation within the suitability range of *P. koraiensis* and provided information relevant to forest management and climate-adaptation strategies. Distribution-based assessments have similarly been used to evaluate climate-change threats to plant diversity [23].

## 3. Results

### 3.1. Potential Spatial Distribution Area of Picea koraiensis and Changes in Response to Future Climate

**Figure 2:**
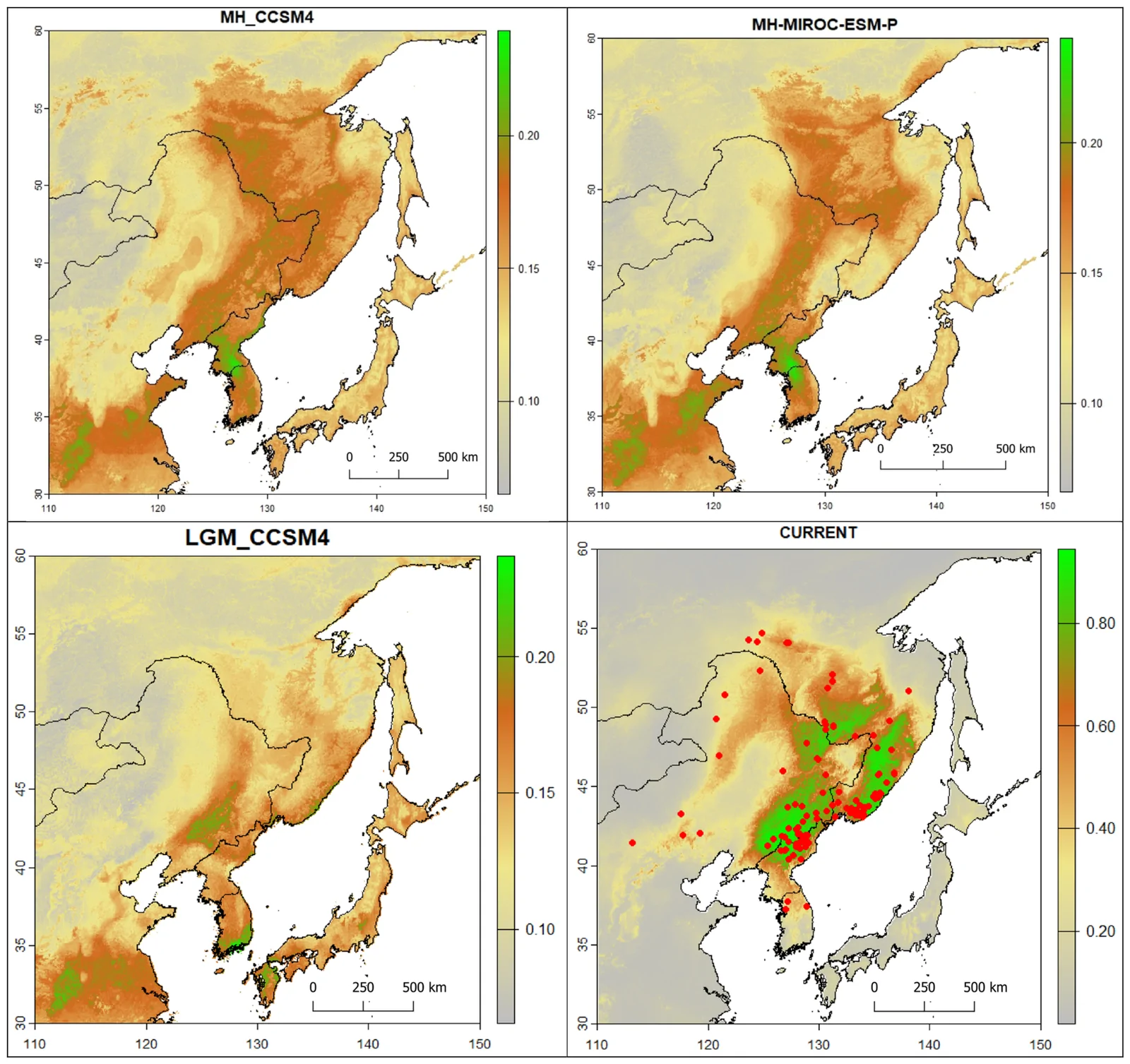

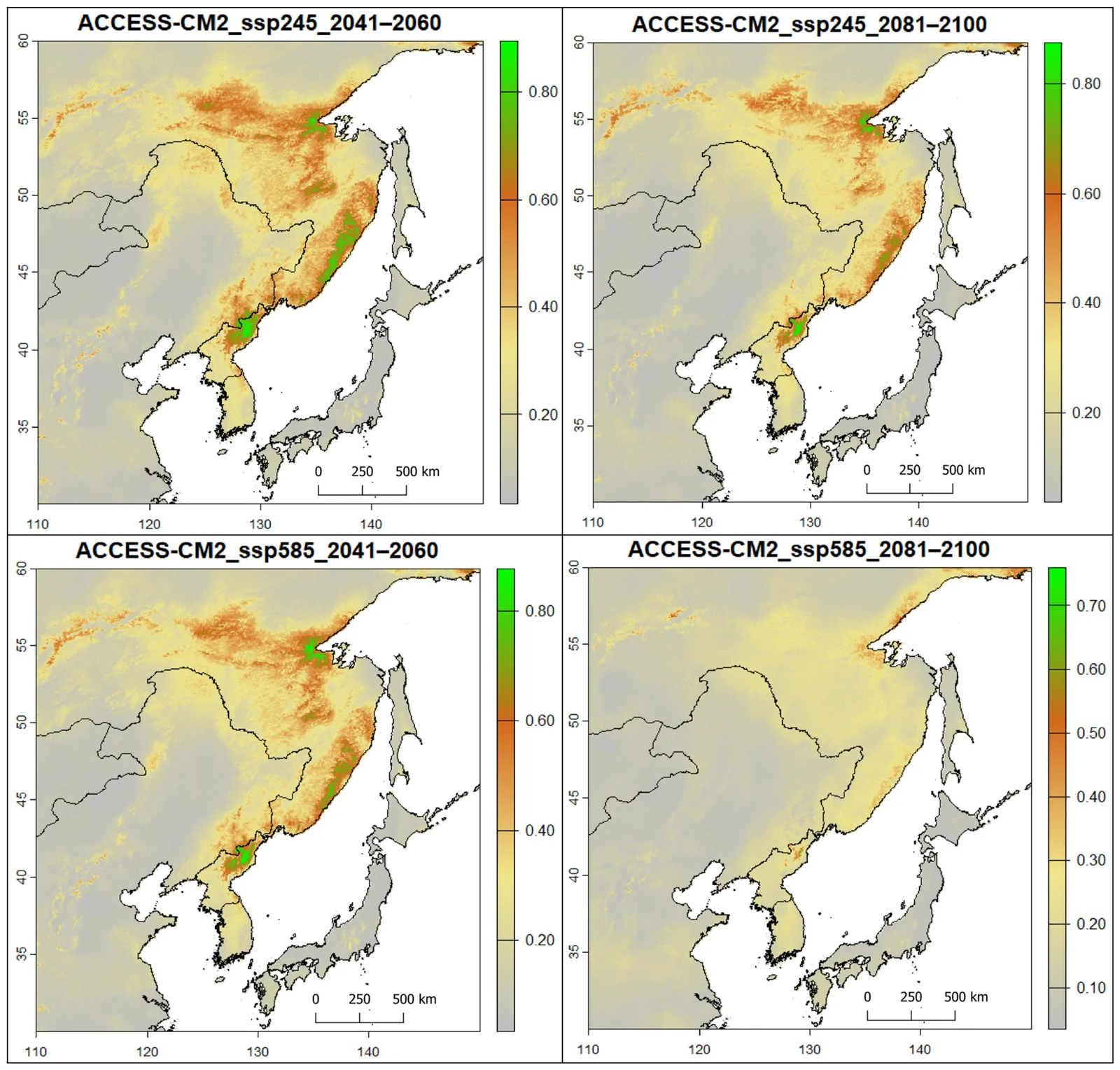
Potential habitat suitability of *Picea koraiensis* at LGM_CCSM4; MH_CCSM4; MH-MIROC-ESM-P; CURRENT and potential shifts under future climate scenarios ACCESS-CM2_ssp245_2041-2060, ACCESS-CM2_ssp245_2081-2100, ACCESS-CM2_ssp585_2041-2060 and ACCESS-CM2_ssp585_2081-2100. The red dots at the CURRENT period represent the occurrence geographic coordinate.

**Figure 3:**
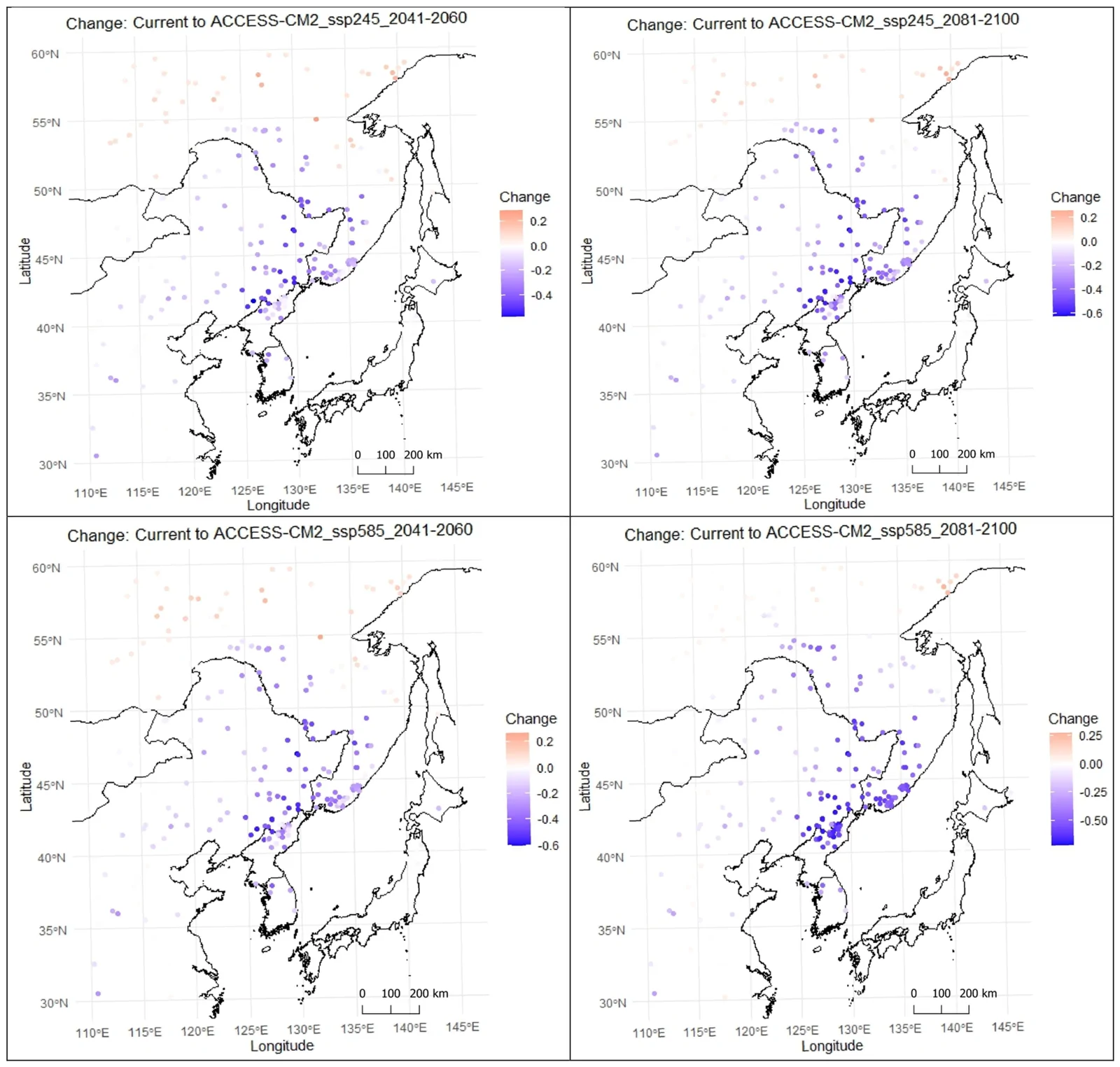
Critical regions predicted to experience the most significant changes for the conservation of *Picea koraiensis*.

Figure 2 shows the predicted areas of *P. koraiensis* over time. Overall, the current range of *P. koraiensis* is larger than that in all other time periods and shows an increase since ancient times and a decrease in the future, especially under the CPRs 585 climate scenarios (Figure 2). Compared with other past climatic conditions and future climate projections, current conditions appear to be more favorable (Figure 3), because *P. koraiensis* colonizes a larger area and its past colonization rate does not exceed 20%, indicating a very low probability of colonization. In the future, the increase in temperate climatic conditions, combined with medium and high atmospheric carbon emissions, leads to a dramatic reduction, particularly under high-emission conditions, whereby almost the entire current distribution area should disappear by 2100. Overall, the predicted distribution ranges help us to understand changes in the distribution range of *P. koraiensis* over time and, in particular, identify future areas suitable for its recolonization.

The areas susceptible to extinction and colonization by *P. koraiensis* in response to climate change, from the LGM to future conditions, are shown in Figure 3. Since ancient times, the colonization areas of *P. koraiensis* have shifted from south to north and are now located in south-eastern Russia. In the next 40–60 years, *P. koraiensis* will occupy only a very small part of the colonizable area in south-eastern Russia, which will require measures to maintain the current range or processes to support colonization. This dynamic illustrates the dual pressure of habitat loss and dispersal under changing environmental conditions. Areas at risk of extinction, particularly in the south or at lower altitudes, reflect rising temperatures and reduced habitat suitability. Areas where the species is colonizing, especially in the north or at higher altitudes, show the species’ potential for adaptation.

### 3.2. Relative Importance of Variables and Response Curves

**Figure 4:**
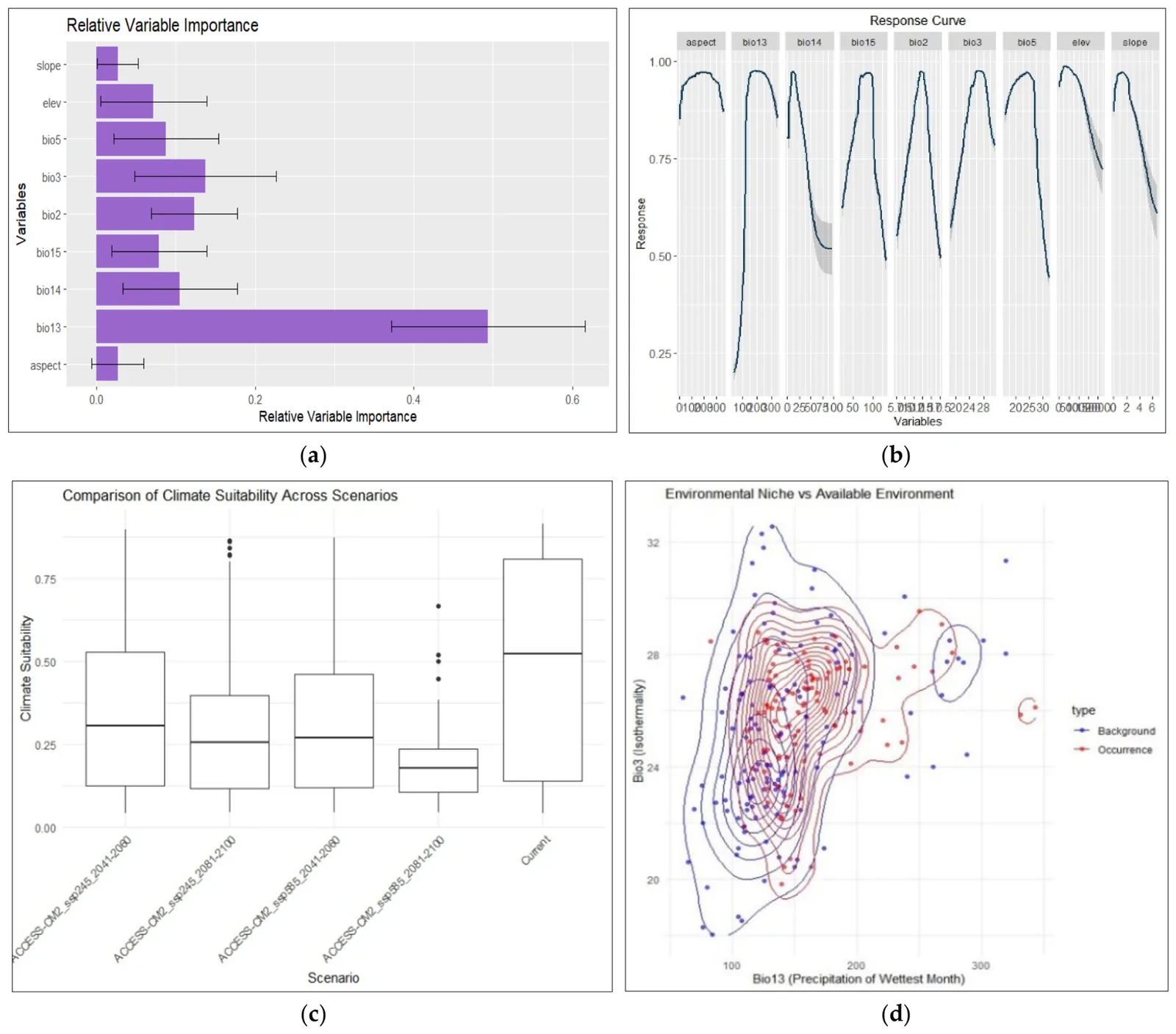
Relative variable importance (**a**), response curves (**b**), climatic suitability across all scenarios (**c**) and Bioclimatic Similarity Curve (**d**) within the distribution range of the most influential bioclimatic factors on the spatial distribution of *Picea koraiensis.* The blue line in Figure 4b shows the probability of presence of *P. koraiensis* following the intensity of the effect of environmental variables and the grey areas represent the 95% confidence interval.

The environmental conditions under which the probability of occurrence of *P. koraiensis* was determined are shown in Figure 4. Based on the correlation and AUC metric, the relative importance of the variables ranged from 2.7% for aspect and slope to 49.4% for bio13. Among the variables with the greatest influence, bio3 accounted for 12.7.1%, bio2 for 12.3%, and bio14 for 10.5%. Based on the AUC metric, bio13 remains the most important variable (39.4%), followed by bio3 (11.7%). In the ensemble model, bio13 and bio3 were the most important variables in predicting the distribution areas of *P. koraiensis*. The ecological niche of *P. koraiensis*, based on the selected key environmental variables under current conditions, showed that *P. koraiensis* requires a certain range: aspect, 150°; bio13, 225 mm; bio14, 10 mm; bio15, 65 mm; bio2, 12 °C; bio3, 27%; bio5, 23 °C; elevation, 450 m; and slope, 1.125° (Figure 4b,d). The suitability of climatic conditions decreases significantly with greenhouse-gas emissions, particularly under SSP5-8.5 scenarios (Figure 3).

### 3.3. Spatial Variation in Key Bioclimatic Variables Within the Distribution Area of Picea koraiensis

**Figure 5:**
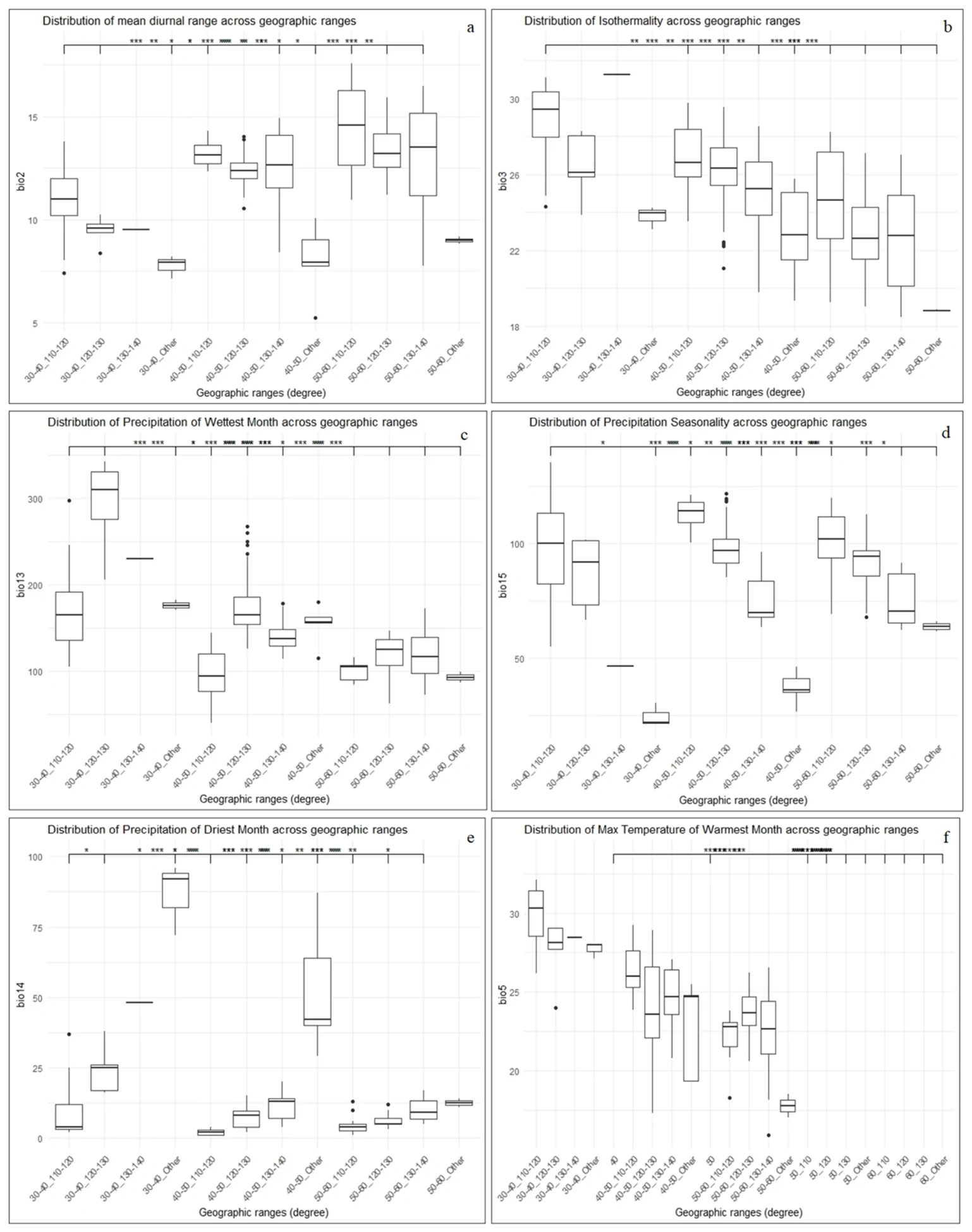
Spatial variation in six key bioclimatic variables across the natural distribution range of *Picea koraiensis*: mean diurnal temperature range (bio2) (**a**), Isothermality (bio3) (**b**), precipitation of the wettest month (bio13) (**c**), precipitation seasonality (bio15) (**d**), precipitation of the driest month (bio14) (**e**), and maximum temperature of the warmest month (bio5) (**f**). Boxplots show the distribution of observed values, with the central line indicating the median, boxes representing the interquartile range, and whiskers showing the dispersion of the data. Asterisks indicate statistically significant differences in mean values, with *, **, and *** denoting increasing levels of statistical significance.

The spatial distributions of bioclimatic variables (bio2, bio3, bio5, bio13, bio14, and bio15) within the species’ range are shown in Figure S3. The culmination zones of the environmental gradients of the selected key bioclimatic variables are located differently in space (Figure S3). The latitudinal and longitudinal variation in the selected key bioclimatic variables (bio2, bio3, bio5, bio13, bio14, and bio15) within the natural distribution area of *P. koraiensis* is shown in Figure 5a–f. Highly significant differences in the latitudinal and longitudinal variation in the selected key bioclimatic variables were observed, especially between 30° and 40° latitude and between 130° and 140° longitude, for all selected bioclimatic variables within the distribution range of *P. koraiensis*, except bio14, which showed slightly significant variation from 110° longitude. Bio5 showed a significant difference only from 50° latitude and between 110° and 120° longitude (Figure 5f).

### 3.4. Spatial Autocorrelation of Key Bioclimatic Variables Within the Distribution Area of Picea koraiensis

**Figure 6:**
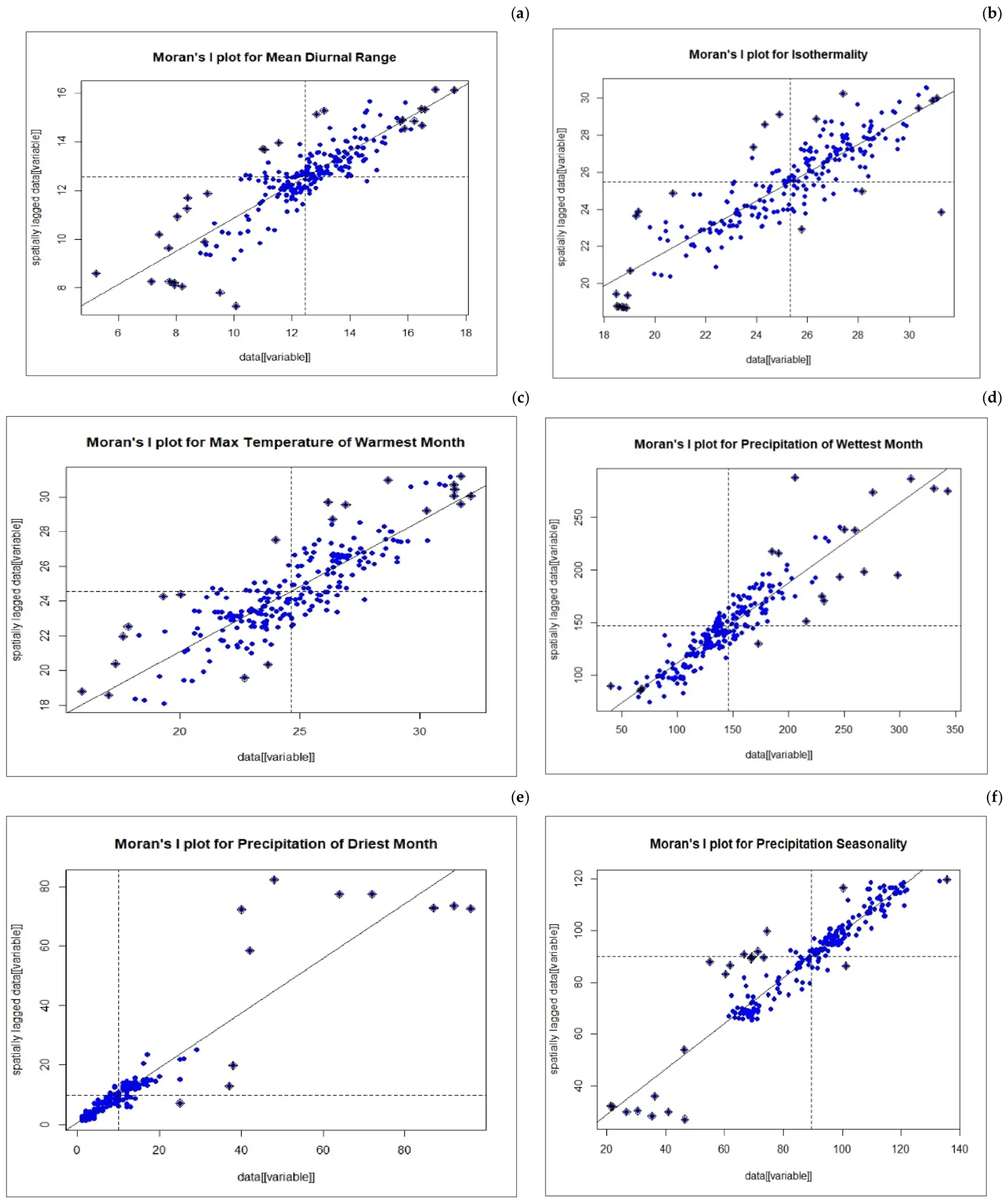
Moran’s I plots for spatial autocorrelation of bioclimatic variables: (**a**) bio2, (**b**) bio3, (**c**) bio5, (**d**) bio13, (**e**) bio14, and (**f**) bio15 across the distribution area of *Picea koraiensis*. The oblique regression line represents the global spatial autocorrelation, with its slope corresponding to Moran’s I. The vertical and horizontal reference lines indicate, respectively, the mean of the observed variable and the mean spatial lag. The four quadrants identify the corresponding spatial clusters and spatial outliers.

The spatial autocorrelation of the bioclimatic variables (bio2, bio3, bio5, bio13, bio14, and bio15) within the species’ distribution area is shown in Figure 6. Overall, all selected key bioclimatic variables showed statistically significant spatial autocorrelation (*p* values < 0.05), indicating a tendency towards similar environmental conditions among occurrence points in different regions within the distribution area of *P. koraiensis*. Moran’s I value ranged from 0.442 to 0.831 (Figure S4), indicating moderate to strong positive spatial autocorrelation for all variables. Precipitation-related variables (bio13, bio14, and bio15) showed the strongest spatial autocorrelation, indicating that precipitation patterns are the most spatially structured factors for the distribution of *P. koraiensis*. Temperature-related variables (bio2, bio3, and bio5) showed moderate to strong spatial autocorrelation, suggesting that temperature patterns also play an important role in the distribution of the species, but have slightly less spatial structure than precipitation. The very strong spatial autocorrelation of bio14 (precipitation of the driest month) suggests that dry-season precipitation may be a crucial factor in determining the spatial distribution of *P. koraiensis*. The high spatial autocorrelation of bio15 (precipitation seasonality) suggests that the seasonal pattern of precipitation is also an important factor in determining the species’ distribution. The spatial patterns of the Local Indicators of Spatial Association (LISA) showed different locations of ecological similarity among the selected bioclimatic variables, except for bio14 and bio15, which indicated similar regions (Figure S5).

### 3.5. Relationship Between Bioclimatic Variables and Terrain Gradients

**Figure 7:**
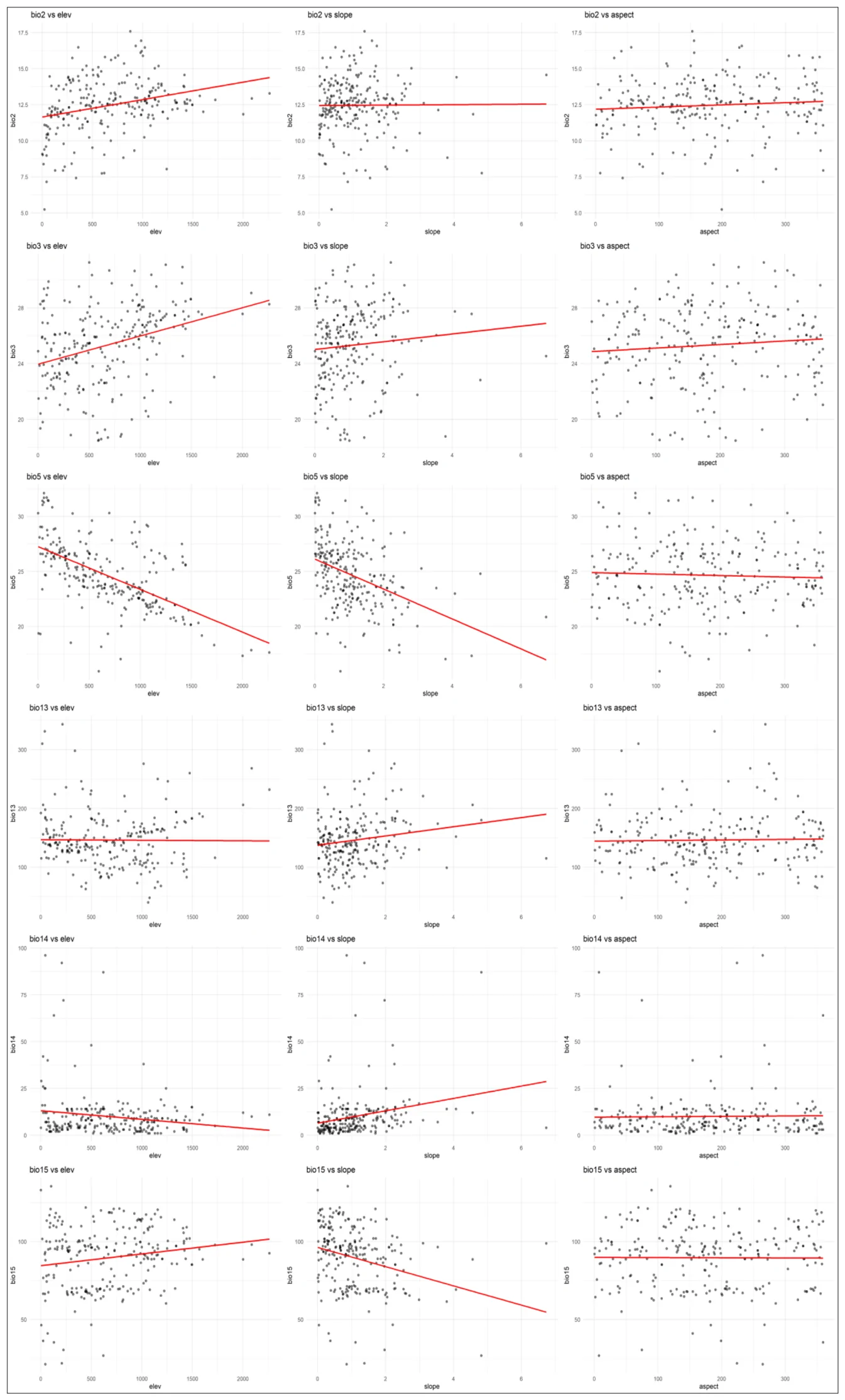
Relationships between selected bioclimatic variables (bio2, bio3, bio5, bio13, bio14, and bio15) and terrain factors (elevation, slope, and aspect) across the distribution range of *Picea koraiensis*. Black points represent observed values, and the red lines indicate the fitted regression trends.

**Figure 8:**
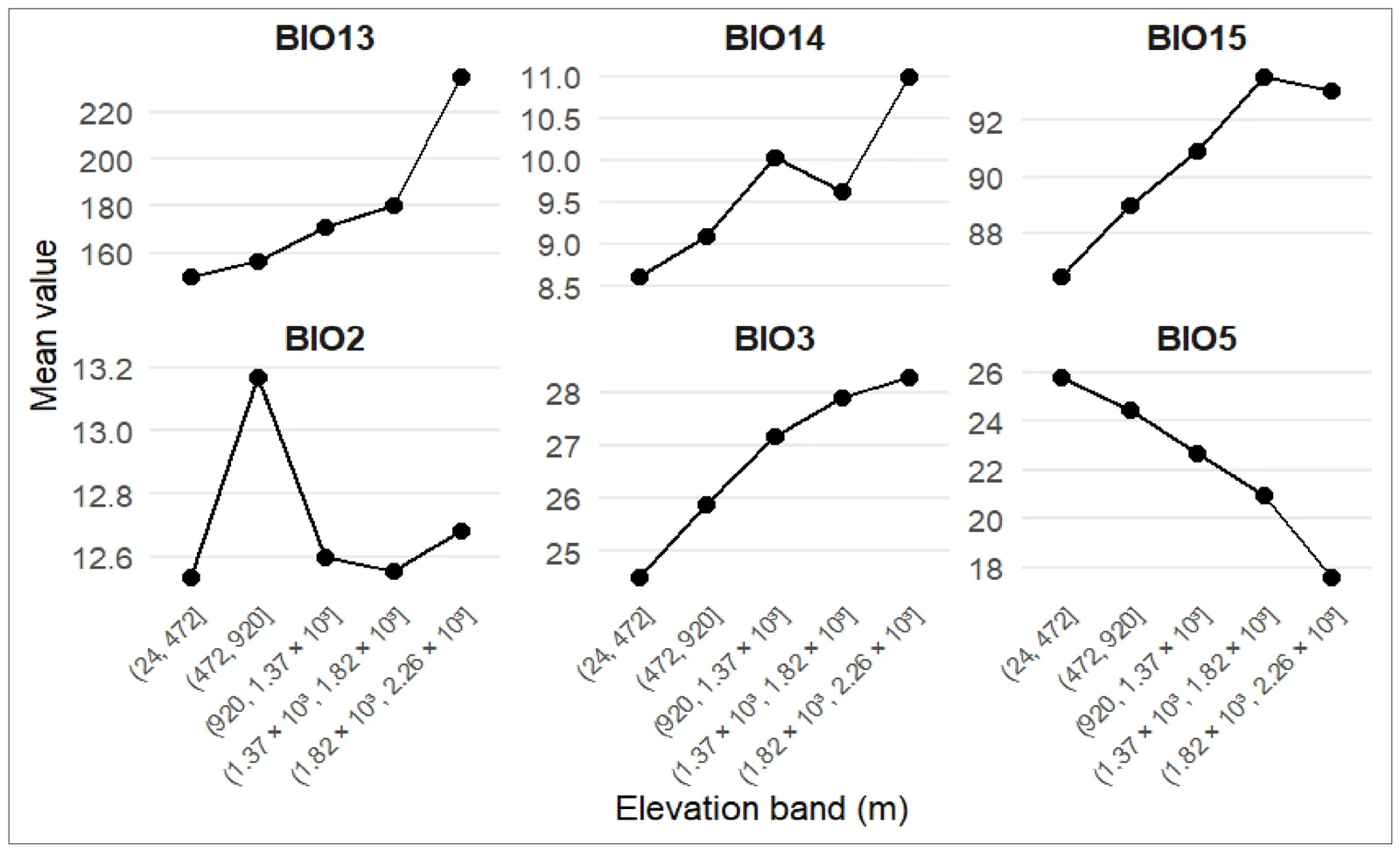
Key bioclimatic variables (bio2, bio3, bio5, bio13, bio14, and bio15) across elevation bands within the distribution range of *Picea koraiensis*.

Multiple linear regression analysis showed that elevation and slope had a significant influence on the selected biological indicators, whereas aspect showed no consistent influence across all models (Figure 7). All selected bioclimatic variables showed specific trends with increasing altitude. Bio3 and bio5 showed completely opposite trends: one increased, whereas the other decreased, with increasing altitude (Figure 8). The intercepts of all models were statistically significant (Table S3), suggesting that factors other than the selected biological indicators also influence these indicators. The generalized additive models (GAMs) for the selected bioclimatic variables in relation to three predictors—elevation, slope, and aspect—showed positive effects for bio3 (isothermality), bio14 (precipitation of the driest month), and bio15 (precipitation dependence). With increasing altitude, the difference between day and night temperatures stabilized, and more precipitation fell during the driest periods at higher altitudes (Figure 7). In terms of precipitation seasonality, higher-altitude areas exhibited stronger precipitation seasonality and clear differences between rainy and dry seasons. Steeper slopes were associated with higher precipitation amounts in both wet and dry periods, and the positive response of precipitation seasonality indicated that steeper slopes exhibited pronounced seasonal changes in precipitation. Negative effects of altitude and slope were observed for bio5 (maximum temperature of the warmest month), indicating that the maximum temperature during the warmest month decreased with increasing altitude. This suggests that higher elevations may provide cooler habitats during peak temperatures, which may be critical for heat-sensitive species, especially in the context of global warming. A negative effect suggests that the difference between day and night temperatures decreases as slope increases.

## 4. Discussion

### 4.1. Spatial Distribution and Changes in Response to Future Climate Conditions

Analyzing the potential spatial distribution of *P. koraiensis* under past, present, and future climatic conditions provides crucial insights into the species’ habitat dynamics and vulnerability to climate change. This temporal comparison reveals both the historical stability of the species’ range and the projected reduction in suitable habitat under future high-emission scenarios. Our results indicate that the current range of *P. koraiensis* is larger than that predicted for other periods, reflecting relatively favorable contemporary climatic conditions. In contrast, climatic conditions during the Last Glacial Maximum (LGM) provided only limited suitable habitat, with predicted occurrence probabilities generally below 20% (Figure 2). This pattern indicates that the relatively cool and humid conditions of the Holocene may have favored the expansion and persistence of suitable habitats for *P. koraiensis* within its natural range [24]. The resulting contrast among the LGM, Holocene, and current periods is interpreted here as a study-specific historical pattern rather than as evidence of a universal post-glacial response.

In the future, however, suitable habitat is projected to decline substantially, particularly under high-emission scenarios. By 2100, much of the current range may become unsuitable as rising temperatures and altered precipitation patterns exceed the species’ climatic tolerance. Habitat contraction is expected to be especially pronounced in southern and low-elevation areas, while potentially suitable areas are predicted to shift northwards and to higher elevations. Although shifts towards cooler regions are widely anticipated in forest management and adaptation literature [25,26], empirical analyses of eastern North American trees have found that many species show range contraction rather than clear northward expansion [24]. Climate change may also alter regional disturbance regimes; modelling in Daxing’anling projects changes in forest burn probability under future climatic conditions [25]. Broad-scale species-distribution projections can also vary among modelling algorithms, so the magnitude and direction of projected shifts should be interpreted with caution [27].

The colonization patterns of *P. koraiensis* further indicate a northward shift in potential distribution, with future suitable habitats concentrated mainly in south-eastern Russia (Figure 3). This projected shift should not be interpreted as evidence that *P. koraiensis* will necessarily track climate successfully, because empirical evidence from other tree assemblages shows that range expansion can lag behind climatic change [24]. However, the predicted colonization capacity of *P. koraiensis* appears limited. Even where future climatic conditions become suitable, successful establishment may be constrained by limited seed dispersal, habitat fragmentation, competition with other species, and low rates of population expansion. Therefore, the projected contraction of suitable habitat in southern areas may not be fully offset by expansion into northern areas. This finding is consistent with broader ecological evidence showing that the ability of tree species to track changing climates depends not only on climatic suitability but also on landscape connectivity, dispersal capacity, and human intervention [28].

Overall, the projected northward and upward range shift in *P. koraiensis* is consistent with the broader expectation that climate change will alter the distributions of boreal and temperate forests. Observed tree-range responses, however, can include contraction without compensating poleward expansion [24]. Species native to the Korean Peninsula and neighboring regions may be particularly sensitive to these changes, with habitat loss expected to accelerate under high-emission scenarios [29]. Moreover, the long generation times and life-history characteristics typical of conifers may slow demographic responses to rapidly changing climates [30]. These results highlight the importance of maintaining habitat connectivity, restoring degraded habitats, and considering assisted migration where appropriate to support the long-term persistence of *P. koraiensis*.

### 4.2. Key Bioclimatic Variables Influencing the Distribution of Picea koraiensis

The most influential predictor was bio13 (precipitation of the wettest month), which contributed 39.4% to the model prediction, followed by bio3 (isothermality; 11.7%). This result highlights the importance of moisture availability during the wettest period and suggests that *P. koraiensis* depends on a relatively stable water supply during critical growth stages. Precipitation variability has also been identified as an important determinant of the distribution of other boreal and temperate tree species [31]. The high contribution of bio13 therefore indicates that future shifts in precipitation regimes may strongly affect the persistence of suitable habitat for *P. koraiensis*. The importance of bio3 further suggests that temperature stability is relevant to the species’ distribution. More broadly, recent climate change has produced coherent biological responses across many natural systems [32]. Bio2 (mean diurnal range) and bio5 (maximum temperature of the warmest month) also contributed to the model prediction, indicating that the species is associated with relatively cool environments with limited temperature variability. In contrast, topographic variables made comparatively small contributions; aspect accounted for 2.7%, whereas slope also had only a minor effect. Nevertheless, these variables may remain relevant at local scales by modifying microclimatic conditions and habitat suitability, particularly in fragmented landscapes.

The ecological niche model under current climatic conditions indicates that *P. koraiensis* is associated with relatively narrow climatic ranges. Suitable conditions were associated with a mean diurnal temperature range of approximately 12 °C (bio2) and a maximum temperature of about 23 °C in the warmest month (bio5) (Figure 4). These conditions are consistent with the cool summers and relatively stable temperature regimes generally associated with boreal species. Future warming may therefore increase physiological stress, especially in low-elevation parts of the species’ range. Precipitation during both the wettest month (bio13, 225 mm) and the driest month (bio14, 10 mm) was also important for habitat suitability. Regional modelling in central Siberia has likewise projected substantial climate-driven changes in forest types and stand heights, with outcomes differing among climate scenarios and moisture conditions [33]. Previous studies have also identified precipitation and temperature as important predictors of tree species distributions in boreal and temperate forests [31]. Broad syntheses document coherent biological responses to recent climate change across natural systems, but do not establish a *P. koraiensis*-specific sensitivity [32]. Accordingly, the contribution of bio3 in the present model suggests that reduced temperature stability under future warming may further constrain the suitable range of this cold-adapted species.

### 4.3. Spatial Variation in Bioclimatic Variables Across the Distribution Area of Picea koraiensis

Spatial variation in bioclimatic variables across the distribution range of *P. koraiensis* provides insight into how environmental gradients influence habitat suitability. These variables represent key aspects of regional temperature and precipitation regimes and are closely linked to the species’ ecological niche. Marked spatial variation was observed in the selected variables, particularly between 30 and 40° latitude and 130 and 140° longitude. Maximum temperature of the warmest month (bio5) and precipitation-related variables, including precipitation of the wettest month (bio13) and precipitation seasonality (bio15), showed pronounced variation along these gradients. In contrast, precipitation of the driest month (bio14) exhibited relatively limited spatial variation, especially east of 110° longitude, indicating comparatively stable dry-season precipitation conditions in these areas.

The variation in bio5 at higher latitudes and between 110° and 120° longitude indicates that extreme summer temperatures may be an important constraint on the distribution of *P. koraiensis*, particularly near the margins of its range. This pattern is consistent with broad evidence of poleward and elevational shifts across natural systems in response to climate change [32]. The pronounced spatial variation in bio13 and bio15 further highlights the importance of moisture availability and precipitation seasonality. More generally, research on tree ecology and biogeography in environmentally heterogeneous forests has shown that both past and present environmental conditions can shape species distributions [34]. Although bio14 varied less across the study area, dry-season precipitation may still influence habitat suitability at local scales. At finer scales, broad climatic layers may not capture the thermal conditions actually experienced within forest microhabitats; comparisons of stationary and organism-based temperature measurements demonstrate that such mismatches can occur [35]. A study of *Larix gmelinii* and *Quercus mongolica* in the Greater Khingan Range likewise showed species-specific differences in projected responses and adaptation strategies under climate change [36]. Overall, the observed patterns suggest that heat stress and precipitation variability are likely to be important climatic constraints on the distribution of *P. koraiensis*. The lack of consistent migration in other tree assemblages also indicates that climatic sensitivity does not necessarily lead to successful range expansion [24]. Such climatic sensitivity supports the expectation that species distributions may shift in response to changing temperature and precipitation regimes [37].

### 4.4. Spatial Autocorrelation of Key Bioclimatic Variables Across the Distribution Area of Picea koraiensis

Analysis of the spatial autocorrelation of key bioclimatic variables across the distribution range of *P. koraiensis* provides insight into the spatial structure of environmental conditions associated with its habitat. Positive Moran’s I values ranging from 0.442 to 0.831 indicate that the selected climatic variables are significantly clustered in space, with similar environmental conditions tending to occur in geographically adjacent areas. These patterns suggest that the distribution of *P. koraiensis* is associated with spatially structured combinations of temperature and precipitation rather than with isolated climatic conditions.

Precipitation-related variables, including bio13, bio14, and bio15, showed the strongest spatial autocorrelation. This finding indicates that precipitation regimes, particularly dry-season precipitation and precipitation seasonality, are spatially structured across the species’ distribution range. Such spatial structure is ecologically relevant because moisture availability and its seasonal distribution may influence habitat suitability, growth, and regeneration. In particular, the clustering of bio14 and bio15 suggests that areas with similar dry-season moisture conditions and precipitation seasonality may provide relatively stable environmental settings for *P. koraiensis*. However, the spatial autocorrelation of these variables should be interpreted as evidence of environmental clustering rather than as direct evidence that any single variable independently determines species occurrence.

Temperature-related variables, including bio2, bio3, and bio5, also showed moderate to strong spatial autocorrelation, although their spatial structure was generally weaker than that of precipitation-related variables. The spatial pattern of bio5, which represents the maximum temperature of the warmest month, suggests that summer heat may contribute to regional differences in habitat suitability. Greater local variation in temperature-related variables may reflect the effects of elevation, terrain, and microclimatic heterogeneity across the species’ range.

The LISA results further showed that bio14 and bio15 had similar local spatial patterns, indicating that some areas share comparable precipitation regimes. These areas may represent potentially stable climatic settings that warrant further consideration in conservation planning, particularly where they overlap with predicted suitable habitats. In contrast, bio2 and bio5 showed more spatially variable local patterns, suggesting that temperature-related conditions may contribute to finer-scale differences in habitat suitability.

The strong spatial structure of precipitation-related variables indicates that moisture regimes are geographically organized across the study area. Species-specific climate responses have also been reported for *Larix gmelinii* and *Quercus mongolica* in the Greater Khingan Range [36]. Fine-scale microclimatic heterogeneity should be considered when interpreting spatial patterns derived from broad climatic layers, because stationary measurements may not represent the conditions experienced within forest microhabitats [35]. Overall, these findings indicate that both precipitation regimes and temperature conditions contribute to the spatial environmental structure relevant to the distribution of *P. koraiensis*.

### 4.5. Relationships Between Bioclimatic Variables and Terrain Gradients

The relationships between bioclimatic variables and terrain gradients, as revealed by multiple linear regression and generalized additive models (GAMs), highlight the importance of elevation and slope in shaping climatic conditions relevant to *P. koraiensis*. Elevation was a significant predictor of several variables, particularly bio3 (isothermality), bio14 (precipitation of the driest month), and bio15 (precipitation seasonality). With increasing elevation, temperature conditions became more stable, precipitation during the driest month increased, and the maximum temperature of the warmest month (bio5) decreased. These patterns suggest that high-elevation areas may provide cooler conditions and greater dry-season moisture availability, which could be particularly important for a species adapted to cool and humid environments. The negative relationship between elevation and bio5 therefore supports the potential role of higher elevations as relatively cool climatic settings during periods of extreme heat.

The positive relationship between elevation and bio14 indicates that higher-elevation areas may retain more favorable moisture conditions during dry periods. In contrast, the positive association with bio15 indicates greater precipitation seasonality, the ecological consequences of which depend on the accompanying moisture regime. Nevertheless, the combination of lower maximum temperatures and greater dry-season precipitation suggests that some high-elevation areas may provide relatively suitable climatic conditions for *P. koraiensis* under warming scenarios. However, high elevations should not automatically be treated as stable refugia, because warming can be amplified with elevation in some mountain regions [38].

Slope also influenced several bioclimatic variables, particularly bio14, bio15, and bio3. Steeper slopes were associated with greater precipitation during the driest month and stronger precipitation seasonality, indicating that slope may modify local moisture conditions. The negative relationship between slope and bio3 further suggests that steeper terrain may be associated with more stable day–night temperature conditions. The mechanism underlying this relationship cannot be determined from the present analysis, and fine-scale thermal conditions may differ from stationary or gridded measurements [35]. These results indicate that slope can create fine-scale climatic heterogeneity, thereby enhancing or limiting local habitat suitability for *P. koraiensis*. In particular, areas with steeper slopes and relatively high dry-season precipitation may represent important local moisture-retaining habitats during drought periods.

In contrast, aspect did not show a consistent effect across the models, suggesting that its influence on the climatic conditions relevant to *P. koraiensis* was weaker than that of elevation and slope. This may reflect the overriding effects of terrain elevation and slope on regional temperature and precipitation patterns. Species-specific responses reported for *Larix gmelinii* and *Quercus mongolica* in the Greater Khingan Range caution against extrapolating terrain effects from one tree species to another [36]. The observed decline in bio5 with elevation is supported directly by the present analysis. These terrain effects should also be interpreted in the context of elevation-dependent climate change, which can accelerate environmental change in mountain ecosystems and produce contrasting regional patterns [38]. Overall, these results demonstrate that the interaction between topography and climate contributes to the spatial heterogeneity of potentially suitable habitats for *P. koraiensis*.

## 5. Conclusions

This study provides important insights into potential climate-driven changes in the distribution of *P. koraiensis*. Although the species currently occupies a relatively large suitable range, future climate scenarios predict a substantial decline in suitable habitat, particularly under high-emission conditions, with up to 80% of the current suitable range potentially lost by 2100. The projected northward and upward shift in suitable habitats towards south-eastern Russia indicates that the species may need to track cooler climatic conditions as temperatures rise. However, the limited dispersal capacity of *P. koraiensis* may constrain its ability to colonize newly suitable areas. Precipitation-related variables, particularly those associated with moisture availability during dry periods, were the strongest predictors of habitat suitability, while terrain factors such as elevation may provide relatively cool climatic refuges. Conservation strategies should therefore prioritize the protection of climatically suitable habitats, especially potential refugia, maintain or restore connectivity corridors, and support migration or assisted establishment where appropriate. Without proactive management, the projected loss and fragmentation of suitable habitat may increase the risk of local population decline or disappearance under accelerating climate change. Future research should integrate long-term monitoring of population dynamics, regeneration, and climate responses across the current and projected range of *P. koraiensis*, together with field-based validation of modelled habitat shifts, to evaluate its adaptive capacity and improve conservation planning under continued climate change.

## Data Availability

The datasets supporting the conclusions of this article are included in the article and its Supplementary Files.

## Supplementary Materials

The following supporting information can be downloaded at https://www.mdpi.com/article/10.3390/biology15171465/s1. Table S1: Description of environmental, bioclimatic, and terrain variables; Table S2: Model algorithm performances; Table S3. Generalized additive models (GAMs) highlighting the importance of elevation and slope in shaping climatic conditions for *Picea koraiensis*; Figure S1: PCA, proportion of variation explained and Variance inflation factor (VIF) on bioclimatic and terrain variable in the distribution area of *Picea koraiensis*; Figure S2: Correlation coefficients among (a) 19 bioclimatic and three terrain variables and (b) the selected uncorrelated variables; Figure S3: Environmental heterogeneity of key bioclimatic variables across the distribution area of *Picea koraiensis*; Figure S4: Spatial autocorrelation Moran’s I coefficients on bioclimatic variables (bio2, bio3, bio5, bio13, bio14, bio15) in the distribution area of *Picea koraiensis*; Figure S5: Spatial patterns of Local Indicators of Spatial Association (LISA) for bioclimatic variables (bio2, bio3, bio5, bio13, bio14, and bio15) across the distribution range of *Picea koraiensis*.
