# Supplementary material for: Spatial Variation of Key Bioclimatic Factors in the Natural Range of Picea koraiensis and Implications for Its Conservation
Source: Biology (Basel). 2026 Aug 28;15(17):1465. doi: 10.3390/biology15171465 (PMC13564778; doi:10.3390/biology15171465)
Supplement: Supplementary file 1 [file biology-15-01465-s001.zip › Supplementary materials Fig S1-S5.pdf]

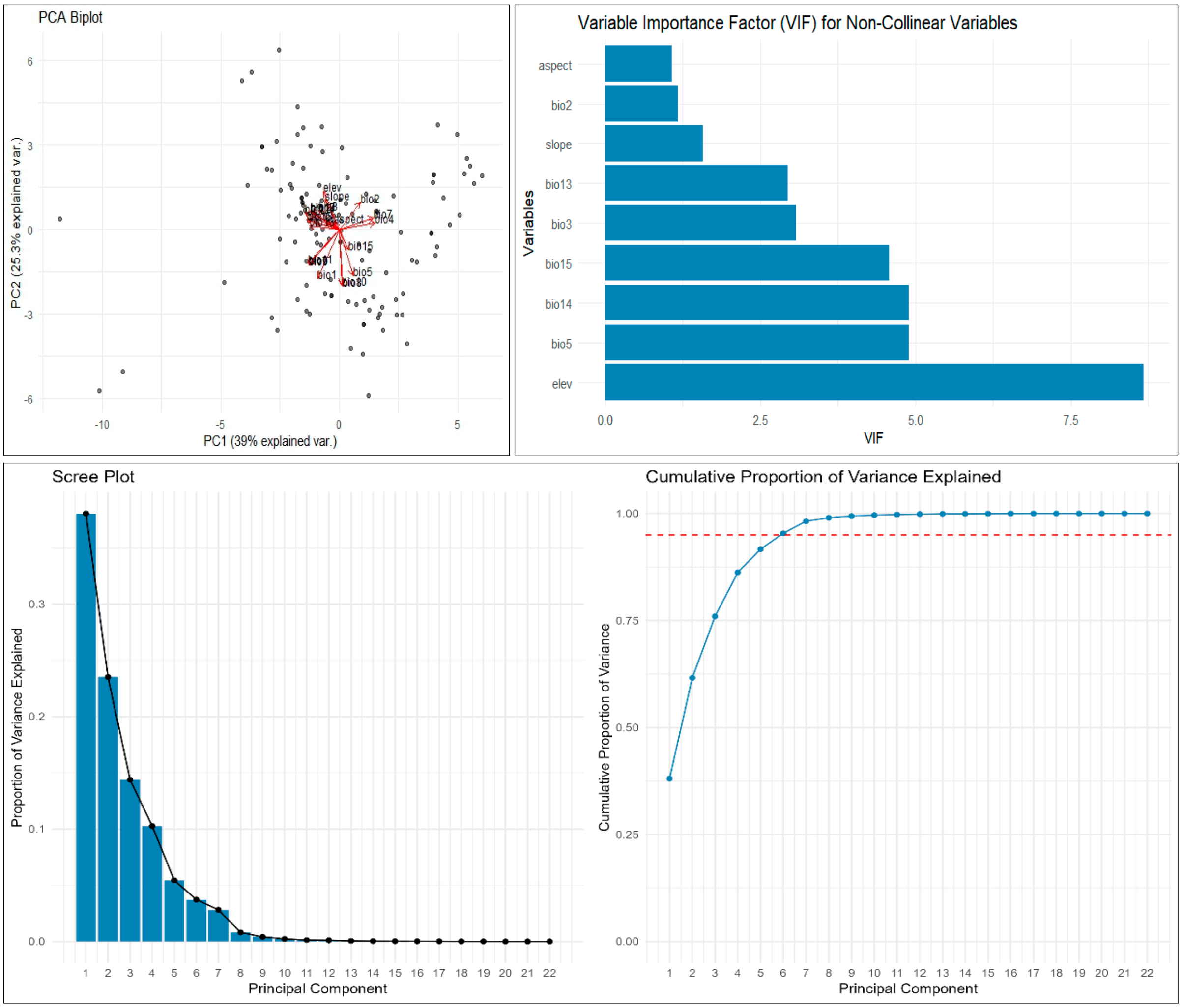

Figure S1: PCA, proportion of variation explained and Variance inflation factor (VIF) on bioclimatic and terrain variable in the distribution area of *Picea koraiensis*

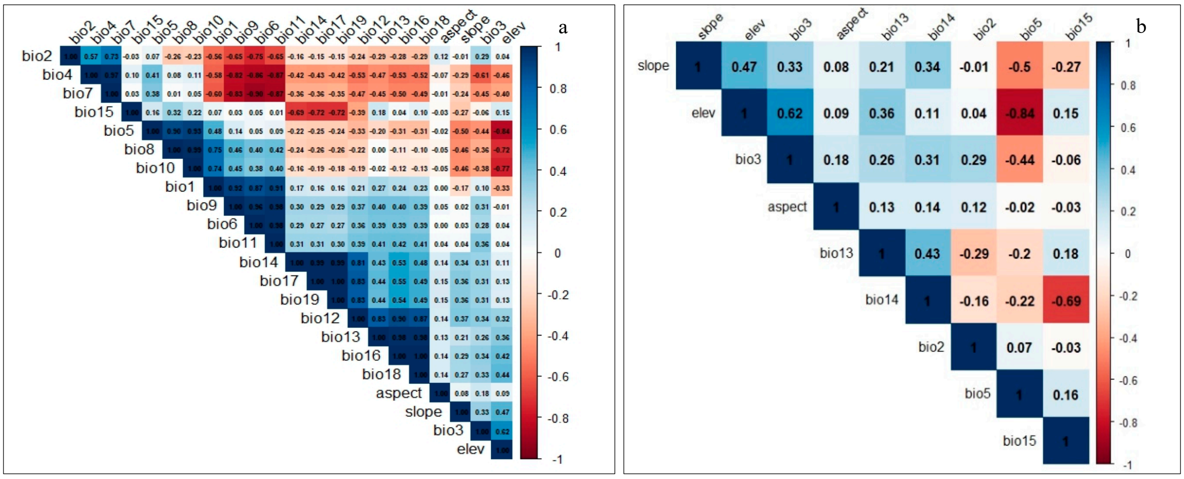

Figure S2: Correlation coefficients between (a)19 bioclimatic and 3 terrain variables and (b) selected uncorrelated variables

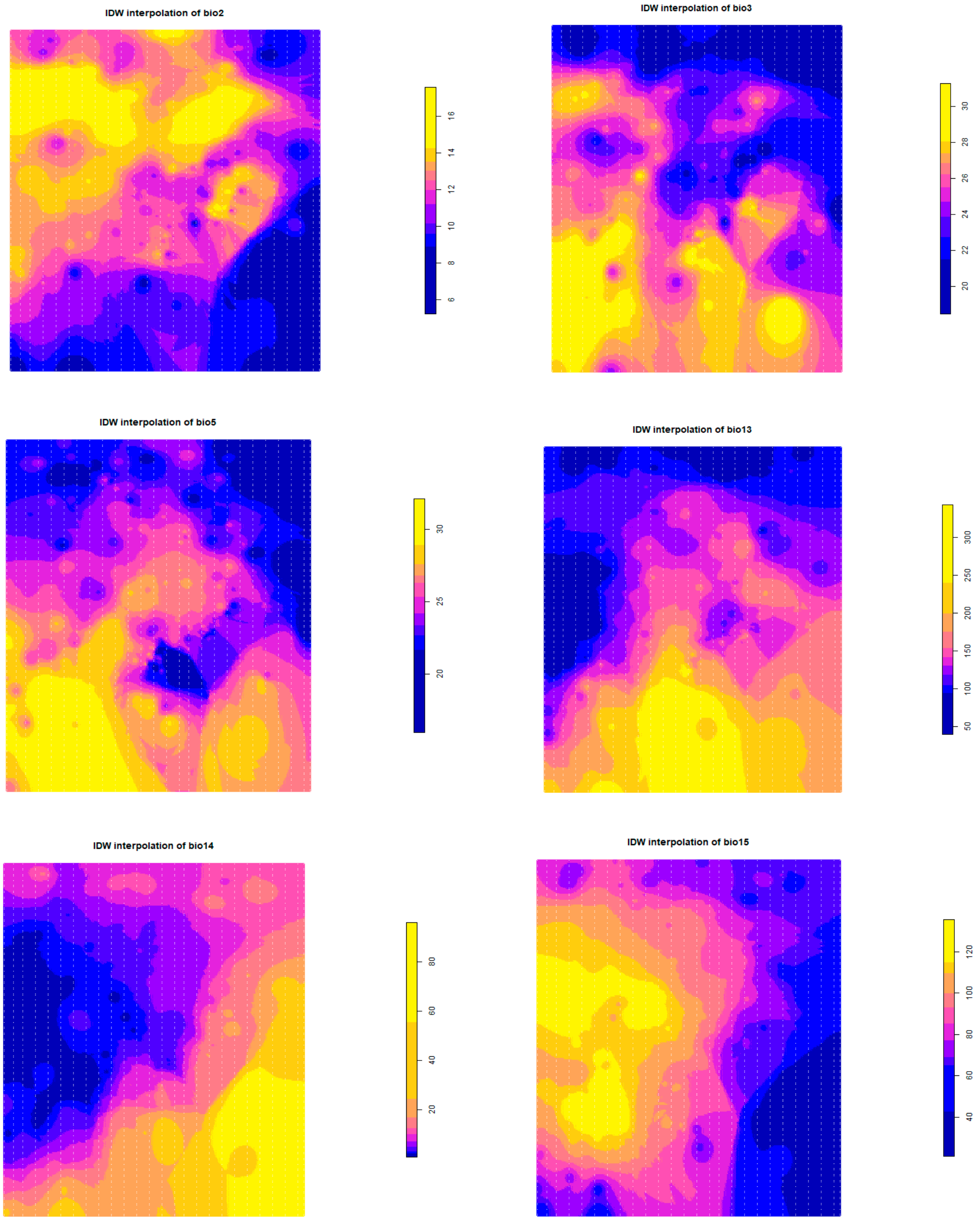

Figure S3: Environmental heterogeneity of key bioclimatic variables in in the distribution area of *Picea koraiensis*

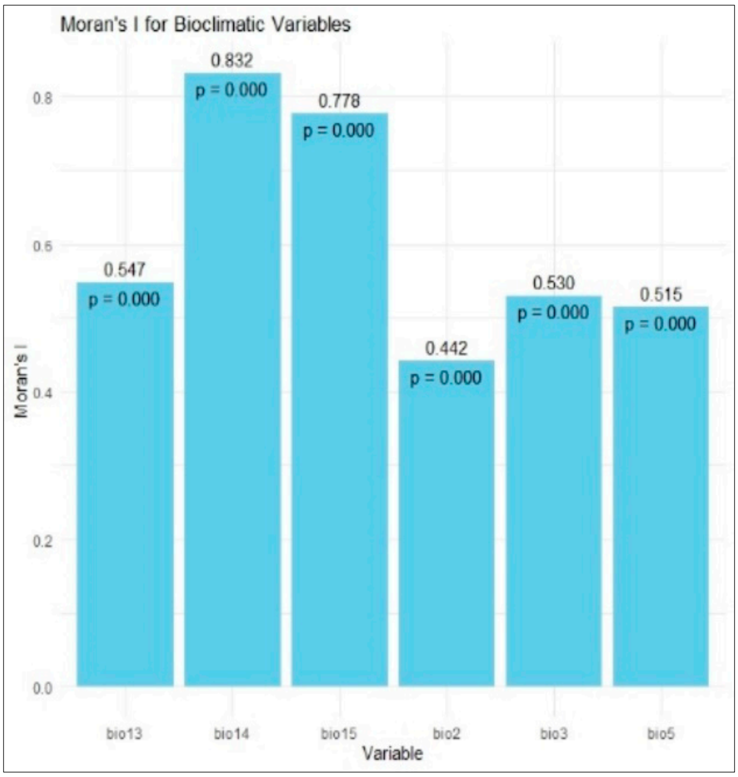

Figure S4: Spatial autocorrelation Moran's I coefficients on bioclimatic variables (bio2, bio3, bio5, bio13, bio14, bio15) in the distribution area of *Picea koraiensis*.

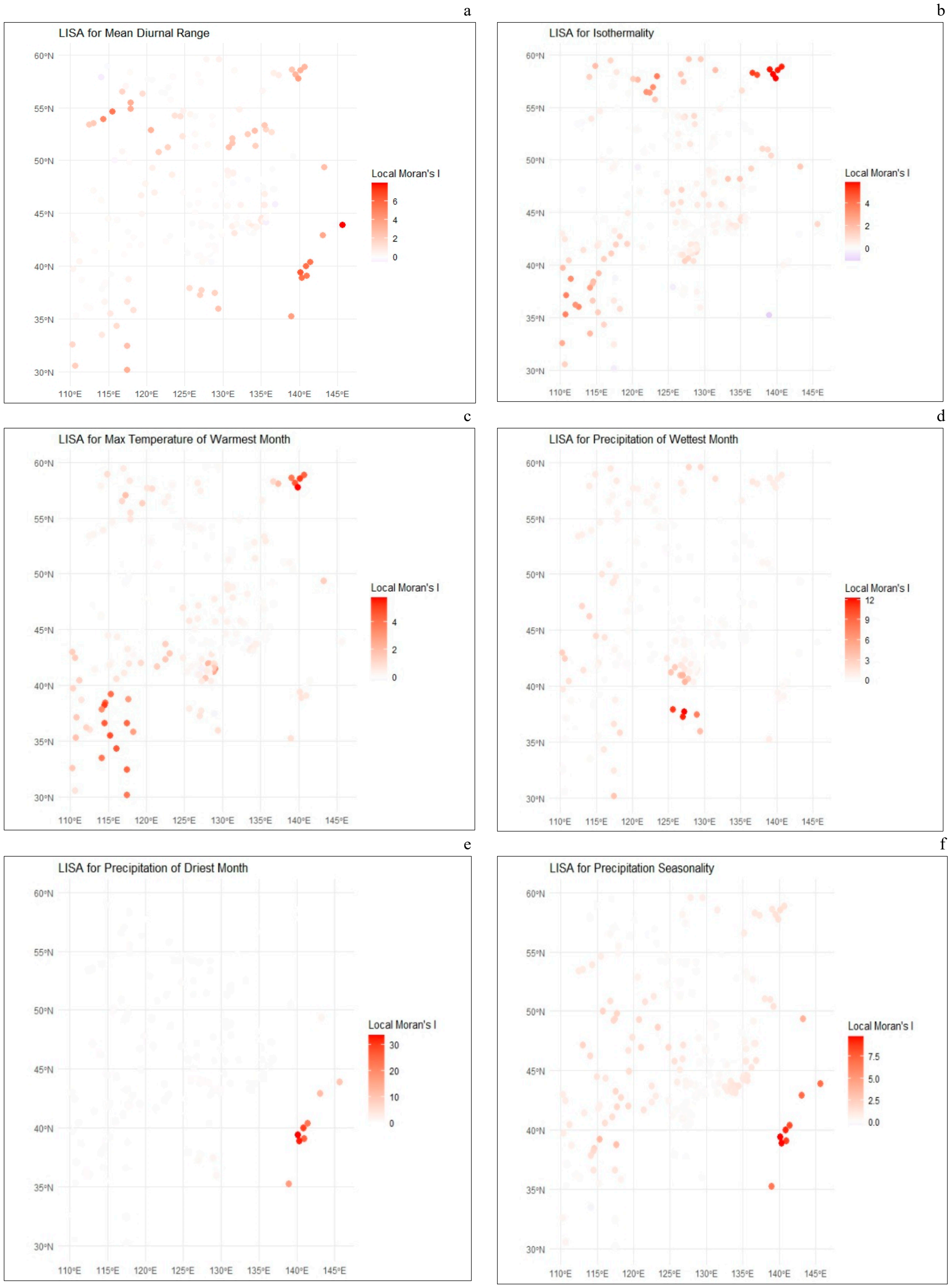

Figure S5: Spatial patterns of Local Indicators of Spatial Association (LISA) in bioclimatic variables (bio2, bio3, bio5, bio13, bio14, bio15) in the distribution range of *Picea koraiensis*.
