# Supplementary material for: Spatial Variation of Key Bioclimatic Factors in the Natural Range of Picea koraiensis and Implications for Its Conservation
Source: Biology (Basel). 2026 Aug 28;15(17):1465. doi: 10.3390/biology15171465 (PMC13564778; doi:10.3390/biology15171465)
Supplement: Supplementary file 1 [file biology-15-01465-s001.zip › Supplementary materials Table S1-S3.pdf]

Table S1: Description of environmental bioclimatic and terrain variables

Variables	Variables complete names	Unite	Min.	Max.	Mean
bio1	Annual Mean Temperature	◦C	-6.57	13.21	1.57
bio2	***Mean Diurnal Range***	***◦C***	***8.40***	***16.48***	***12.69***
bio3	***Isothermality (Bio2/Bio7) (*100)***	***%***	***19.81***	***47.67***	***26.10***
bio4	Temperature seasonality	%	478.55	1817.36	1347.46
**bio5**	**Max Temperature of Warmest Month**	**◦C**	**17.33**	**29.04**	**24.10**
bio6	Min Temperature of Coldest Month	◦C	-38.64	-0.82	-24.92
bio7	Temperature Annual Range	◦C	24.68	64.86	49.02
bio8	Mean Temperature of Wettest Quarter	◦C	10.54	23.43	17.01
bio9	Mean Temperature of Driest Quarter	◦C	-27.68	7.58	-15.79
bio10	Mean Temperature of Warmest Quarter	◦C	10.54	23.87	17.20
bio11	Mean Temperature of Coldest Quarter	◦C	-27.70	7.00	-16.03
bio12	Annual Precipitation	mm	315.00	1371.00	727.26
bio13	***Precipitation of Wettest Month***	***mm***	***83.00***	***343.00***	***160.16***
bio14	***Precipitation of Driest Month***	***mm***	***1.00***	***38.00***	***9.23***
bio15	***Precipitation Seasonality***	***mm***	***63.37***	***119.66***	***88.46***
bio16	Precipitation of Wettest Quarter	mm	211.00	799.00	415.69
bio17	Precipitation of Driest Quarter	mm	5.00	128.00	34.32
bio18	Precipitation of Warmest Quarter	mm	211.00	784.00	411.19
bio19	Precipitation of Coldest Quarter	mm	5.00	128.00	34.49
aspect	Aspect	◦	0.55	356.97	178.36
slope	Slope	◦	0.01	4.57	1.18
elev	Elevation	m	24.00	2744.00	735.62

Selected variables are in bold and italic

Table S2: Model algorithms performances

Methods	AUC	COR	TSS	Deviance
bioclim	0.75	0.46	0.49	2.99
bioclim.dismo	0.83	0.5	0.6	2.9
cart	0.86	0.67	0.68	1.37
domain.dismo	0.76	0.42	0.48	1.24
rf	***0.94***	***0.78***	***0.8***	***0.67***
brt	0.9	0.71	0.72	0.95
gam	0.86	0.67	0.71	5.46
glm	0.81	0.56	0.6	1.1
fda	0.79	0.51	0.55	1.13
mda	0.86	0.63	0.63	1.05
glmnet	0.78	0.44	0.52	2.95
glmpoly	0.85	0.63	0.64	3.48
mahal.dismo	0.88	0.27	0.67	4.49
mars	0.88	0.68	0.7	1.59
maxlike	0.72	0.45	0.46	5.93
mexNet	0.91	0.72	0.73	0.85
mlp	0.88	0.67	0.7	1.33
ranger	0.8	NaN	0.66	NaN
rbf	0.88	0.64	0.69	0.93
rpart	0.86	0.64	0.64	1.24
svm	***0.92***	***0.77***	***0.77***	***0.69***
maxent	***0.93***	***0.75***	***0.73***	***0.81***

Selected algorithms are in bold and italic

Table S3: Generalized additive models (GAMs) highlights the importance of elevation and slope in shaping climatic conditions for Picea coraiensis

Term	estimate	std.error	statistic	p.value	climate_var
(Intercept)	11.63	0.28	41.32	0.000	bio2
elev	0.00	0.00	5.06	0.000	bio2
slope	-0.28	0.14	-2.06	0.040	bio2
aspect	0.00	0.00	0.87	0.386	bio2
(Intercept)	23.80	0.41	57.50	0.000	bio3
elev	0.00	0.00	5.15	0.000	bio3
slope	-0.17	0.20	-0.83	0.405	bio3
aspect	0.00	0.00	0.91	0.361	bio3
(Intercept)	27.45	0.37	73.77	0.000	bio5
elev	0.00	0.00	-8.97	0.000	bio5
slope	-0.70	0.18	-3.83	0.000	bio5
aspect	0.00	0.00	0.69	0.489	bio5
(Intercept)	140.62	7.12	19.76	0.000	bio13
elev	-0.01	0.01	-1.29	0.198	bio13
slope	9.59	3.47	2.76	0.006	bio13
aspect	0.01	0.03	0.24	0.810	bio13
(Intercept)	10.06	1.86	5.39	0.000	bio14
elev	-0.01	0.00	-4.84	0.000	bio14
slope	5.10	0.91	5.60	0.000	bio14
aspect	0.00	0.01	0.37	0.713	bio14
(Intercept)	89.70	2.89	31.07	0.000	bio15
elev	0.02	0.00	5.34	0.000	bio15
slope	-9.27	1.41	-6.58	0.000	bio15
aspect	0.00	0.01	-0.16	0.872	bio15
